# Correlation Between Microbial Community and Hatching Failure in Loggerhead Sea Turtle *Caretta caretta*

**DOI:** 10.1007/s00248-023-02197-8

**Published:** 2023-02-20

**Authors:** Fanny Claire Capri, Elena Prazzi, Giulia Casamento, Delia Gambino, Giovanni Cassata, Rosa Alduina

**Affiliations:** 1 Dipartimento Scienze e Tecnologie Biologiche, Chimiche e Farmaceutiche, Viale delle Scienze, University of Palermo, 90133 Palermo, Italy; 2 Legambiente Sicilia-Ente Gestore Riserva Naturale Orientata Isola di Lampedusa, Via Vittorio Emanuele, 25, 92031 Lampedusa, AG Italy; 3 Legambiente Sicilia-Ente Gestore Riserve Naturali, via Paolo Gili,4, 90138 Palermo, PA Italy; 4 Istituto Zooprofilattico Sperimentale della Sicilia “A. Mirri”, Via G. Marinuzzi, 3, 90129 Palermo, Italy; 5 NBFC, National Biodiversity Future Center, Piazza Marina 61, 90133 Palermo, Italy

**Keywords:** Microbiota, 16S rRNA gene metabarcoding, *Caretta caretta*, Nest hatching, *Brucella*, *Pseudomonas*

## Abstract

Microbial communities provide essential information about host ecology and could be helpful as a tool to improve species conservation efforts. However, microbes can also infect and compromise the host development process and viability. *Caretta caretta* is the most widespread marine turtle species in the Mediterranean basin and is the only species of sea turtle nesting along the Italian coasts. Little is known about the microbiota composition of the nest of sea turtles and its correlation with hatching failures. In this study, the microbial composition of two nests of *C. caretta* featuring different rates of hatching success from a nesting beach in Lampedusa (Italy) was analyzed and compared. The bacterial community was determined using culture-dependent methods and next-generation sequencing based on 16S rRNA gene metabarcoding analysis. Our results showed five dominant bacterial phyla (Proteobacteria, Bacteroidetes, Actinobacteria, Verrucomicrobia, and Firmicutes) and indicated different bacterial families (*Pseudomonadaceae* and *Brucellaceae*) as likely causes of hatching failures. Besides, our findings demonstrated the nests’ active role in modulating the sand’s bacterial communities. This study suggests microbiological analysis could be a valuable tool in monitoring nests to take preventive actions and reduce hatching failures.

**Supplementary Information:**

The online version contains supplementary material available at 10.1007/s00248-023-02197-8.

## Introduction

The sea turtle *Caretta caretta* is the most widespread marine turtle species in the Mediterranean basin and is the only species of sea turtle nesting along the Italian coasts. In the last years, the coastal zones of Italy (i.e., the south zones and the islands) have registered an increase in nesting cases. The assessment of new nesting sites in the Pelagian Islands (i.e., Linosa and Lampedusa) highlighted how *C. caretta’s* nesting has occurred over the last 40 years [1, 2].

**Fig. 1:**
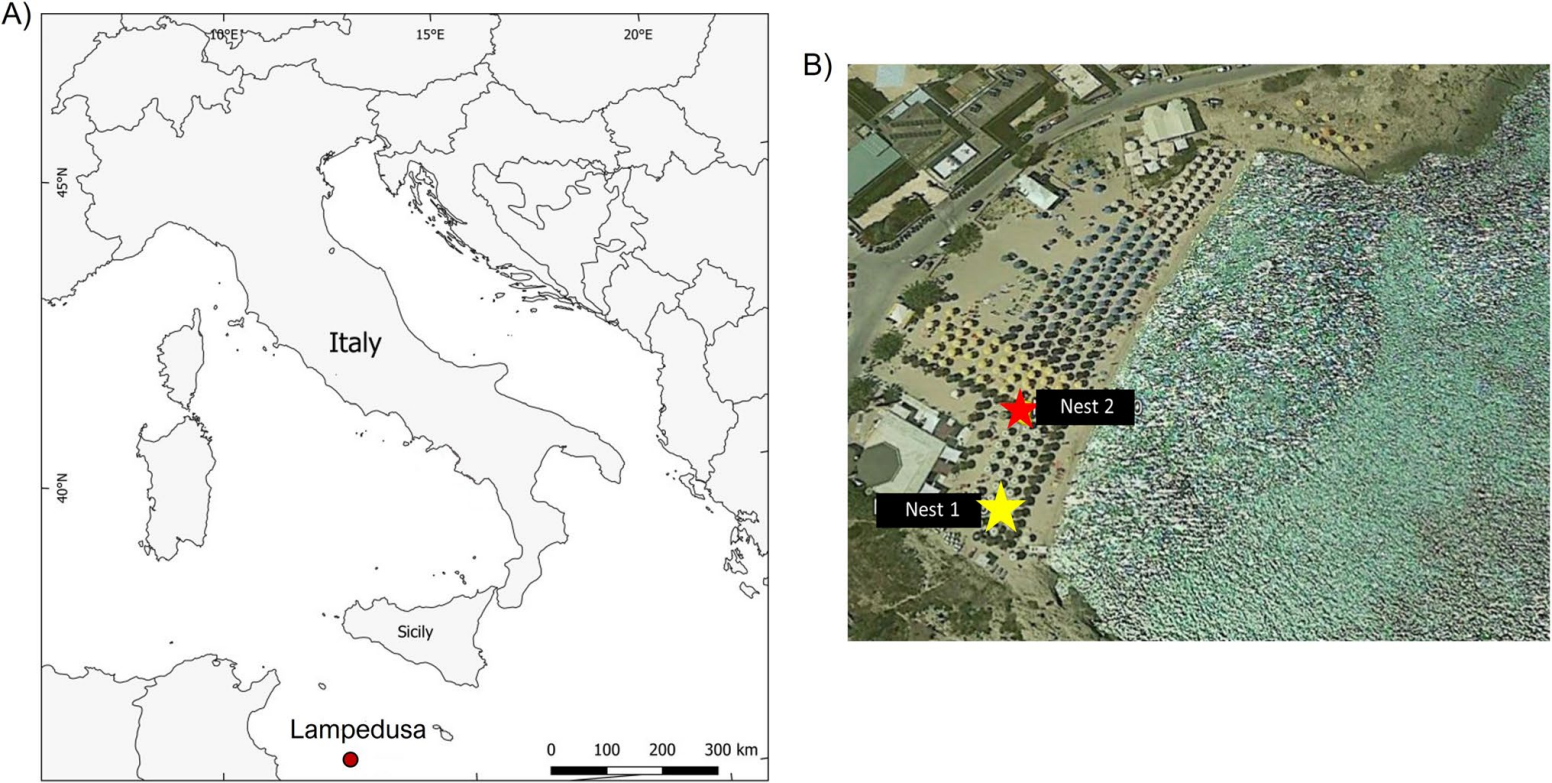
*C. caretta* nests in Lampedusa Island at Guitgia beach. **A** Map and position of Lampedusa Island, indicated by a red circle. **B** The yellow and red stars indicate nests 1 and 2, respectively

According to the International Union for Conservation of Nature (IUCN), *C. caretta* is considered a species of least concern [3]. Many factors, particularly anthropic, threaten the conservation of this species during the juvenile and adult ages at sea and the spawning period and embryonic development in coastal nesting areas. The sea and beach pollution, overbuilding, and degradation of the coastal areas, tourism, climate change, presence of predators, rains, floods, and microbial infections are compromising factors for both hatching and survival rates of the newborn [1–10]). To date, there is an increasing interest in microbial community studies to provide information on the ecology of the host and improve conservation efforts, rescue, and rehabilitation practices. Culture-dependent methods led to the isolation of pathogens from adults [11–13] and nests [9, 14] of *C. caretta* turtles of the Mediterranean Sea. Various bacterial and fungal agents have been described as the major causes of the failure of hatching phenomena of sea turtle eggs, as they can penetrate the outer layer of the eggshell, exploiting embryonic tissues as a source of nutrients [15–17]. Among bacteria, the most representatives belong to the genera *Pseudomonas*, *Vibrio*, *Escherichia*, *Klebsiella*, *Enterobacter*, *Aeromonas*, and *Salmonella*, while *Fusarium* is mostly considered the fungus responsible for infection of sea turtle eggs and nests [9, 14, 18]. In recent years, studies on microbial communities by next-generation sequencing (NGS) of 16S rRNA gene have been applied to the study of the cloacal, oral, intestinal, and skin microbiota of *C. caretta* sea turtles in the Mediterranean Sea [19–23]. Regarding nest microbiota, only a study has been carried out by analyzing metagenomic DNA [24], and it was suggested that the maternal and environmental influence alongside a protective role of eggshells shape the egg microbiota of *C. caretta* sea turtles, as found in other turtles [25, 26]. In the summer of 2020, two *C. caretta*’s nests located at the beach of Guitgia (Lampedusa, Italy) featured different hatching success rates (0% for nest 1 and 58.90% for nest 2). Guitgia beach, extending for about 4.500 m^2^, is located in the southeastern part of Lampedusa Island near the downtown and the port (Fig. 1). Although *C. caretta*’s oviposition has been ascertained since 2018 [27], it is the beach most affected by potential disturbing factors related to mass tourism and flooding phenomena due to rainwater that pours from the surrounding roads onto the beach during heavy rain seasons. The aim of this study was to investigate and compare the microbial composition of the two nests by classical microbiological methods and by 16S rRNA gene metabarcoding to get insights into the failure reasons of *C. caretta* hatchings.

## Experimental Procedures

### Site Description and Sampling

Samples were collected from two nests of the sea turtle *C. caretta* on the beach of Guitgia (35°29′55.2″N, 12°35′57.3″E) at Lampedusa Island (Sicily, Italy) during the summer of 2020 under the authorization from n. 0006828 04/04/2018 for the years 2018–2020 of the Ministero dell’Ambiente e della tutela del Territorio e del Mare. The two nests were 20 m apart. Eighty of 80 eggs were found unhatched in nest 1; 30/73 unhatched eggs were found (58.9% success rate of hatching) in nest 2 (Table 1).

**Table 1:** Description of the sampled pools analyzed in this study

Nest	Distance from the sea	Number of unhatched eggs	Hatching success (%)	Number of metagenomic DNAs	Pools
	10 m			4	Sand outside the nests
1	9.2 m	80/80	0	4	Sand inside the nest
				4	Shells of unhatched eggs
				4	Inner membranes of unhatched eggs
2	15 m	30/73	58.90	4	Sand inside the nest
				4	Shells of unhatched eggs
				4	Inner membranes of unhatched eggs
				4	Shells of hatched eggs

About 72 h after the last hatchling emerged from nest 2, the nests were dug and examined. Four unhatched eggs from nest 1 and four unhatched eggs and fragments of eggshells of hatched eggs from nest 2 were collected together with four sand samples at a depth of 50 cm from both nests. Four samples of external sand were also collected as a reference. All samples were placed in separate sterile bags and kept at 4 °C until stored at − 20 °C in the laboratory. Before analysis, the egg surface was washed with 15 ml of distilled water to remove loosely attached biofilm. The shell of unhatched eggs was opened aseptically with a sterile scalpel, and the inner membrane was separated using sterilized pliers and transferred into sterile tubes. The details of the analyzed samples are summarized in Table 1.

### Bacteriological and Mycological Analyses

Swabs from the eggshells and the internal content of unhatched eggs were taken to isolate bacterial and fungal pathogens. Approximately 1 g of sand samples was incubated in a liquid medium at 37 °C overnight, and 0.1 ml was streaked on agar plates. Three solid culture media were used: blood agar to isolate hemolytic fastidious and non-fastidious bacterial strains, MacConkey agar to select Gram-negative bacteria, thiosulfate-citrate-bile-sucrose agar to isolate *Vibrio*, tryptone soya agar to isolate environmental strains and Sabouraud dextrose agar to allow the growth of the fungus *Fusarium*. For bacterial and fungal growth, plates were incubated at 30 °C for 24–48 h and at room temperature for 7 days, respectively. Bacterial colonies were distinguished based on their morphology and subsequently streaked onto fresh plates. As the growth of suspected fungi was visible, a portion of hyphae was collected with a sterile scalpel and used for further analysis and molecular identification.

### Molecular Identification of Bacteria and Fungi

The identification of bacterial and fungal isolates was carried out by a molecular approach. DNA was extracted and purified by using the phenol-chloroform extraction reported in [28] from bacterial and fungal suspensions grown for 24 h (bacteria) and 48–72 h (fungi) in agitation (180 rpm) at 30 °C in 10 ml of liquid medium Luria-Bertani (10 g tryptone, 5 g yeast extract, and 10 g NaCl, pH 7.0 ± 0.2, autoclaved for 15 min at 121 °C). Bacterial DNA was used as a template in a PCR reaction to amplify the 16S rRNA gene (1500 bp), using the primers F1 (GAGTTTGATCCTGGCTCAG) and R12 (ACGGCTACCTTGTTACGACT) [29]. Fungal DNA was used to amplify a 600-bp internal fragment of the ITS gene using the primer pair ITS-1 (TCCGTAGGTGAACCTGCGG) and ITS-4 (TCCTCCGCTTATTGATATGC) [30]. The presence of the 16S and ITS amplicons was verified by electrophoresis on 1% w/v agarose gel. The amplicons were purified using the QIAquick PCR Purification Kit (Qiagen, West Sussex, UK) according to the manufacturer’s instructions and quantified at the NanoDrop 2000c spectrophotometer (Thermo Fisher Scientific, MA, USA). The amplicons were sequenced by the Sanger method at BMR Genomics s.r.l. (Padova, Italy). The sequence outputs were analyzed using the alignment tool (BLAST). The sequence dataset was deposited in the GenBank database (OM857961-OM857964; OM860305-OM860307; OM860310, OM860311; OM860313-OM860315; OM860317; OM860320; OM860321).

### 16S rRNA Gene Metabarcoding

Samples listed in Table 1 were subjected to metagenomic DNA extraction following the protocol reported in [20] with minor modifications. Sand samples and inner membranes (1 g) were incubated in 1 ml of sterile water for 1 h at room temperature (500 rpm). Small fragments of the shell of hatched and unhatched eggs (of the same size as a 50 ml tube stopper) were homogenized by vortexing in 3 ml of sterile water using sterile glass beads and stirred for 1 h at room temperature. Following this incubation step, samples were processed following the protocol. Metagenomic DNA was verified by electrophoresis on 1% w/v agarose gel. The purity and quantity of DNA were assessed using a NanoDrop 2000c spectrophotometer (Thermo Fisher Scientific, MA, USA). An equal amount of the four extracted metagenomic DNAs from each type of samples was pooled. Thus, a total of 8 metagenomic DNA pools were obtained. A 464-bp fragment corresponding to the V3–V4 region of 16S rRNA gene was amplified using the primers Pro341-F (CCTACGGGNBGCASCAG) and Pro805R (GACTACNVGGGTATCTAATCC) [31]. Amplification products were sequenced in one 300-bp paired-end run on an Illumina MiSeq platform at BMR Genomics s.r.l. (Padova, Italy). The raw 16S rDNA data were processed by using the QIIME2 environment [32] as paired-end sequences. In the denoising approach, overlapping paired-end reads were processed with the plug-in DADA2 [33]. Unique Amplicon Sequence Variants (ASVs) were assigned and aligned to the Greengenes reference database at 99% sequence similarity (https://greengenes.secondgenome.com/). QIIME2 was used to generate rarefaction curves, Good’s coverage index, and alpha diversity metrics (Fig. S1 and Table S1, Supplementary Material). Rarefaction analysis was carried out by plotting the number of the observed ASVs against the total number of filtered reads for each sample. The number of ASVs and the percentage of the relative abundances of different taxonomic levels were determined. Based on the rarefaction curve, the alpha diversity metrics were calculated on a rarefied frequency-feature table with a minimum number of 13,965 sequences per sample. The sequence dataset was deposited in the GenBank database (BioProjectID: PRJNA804141).

## Results and Discussion

### Presence of *Fusarium* spp.

The search for the presence of *Fusarium* in all our samples showed that, independently from the nest, two genera of *Fusarium*, i.e., *F. solani* and *F. falciforme*, were present. According to several reports [9, 15–17], fungi of the genus *Fusarium* were recognized as primary causes of death and reduced hatching success rates in sea turtles. Although the shell represents a protective barrier from the outer environment, it does not completely inhibit the passage of fungi, which produce lipolytic and proteolytic enzymes and penetrate the shell layers, causing the reduction of respiratory gas exchange, decreasing the availability of calcium for developing embryos, and using developing tissues as nutrient sources [34]. Specifically, *F. solani* was isolated from the shell of unhatched eggs of nest 1 and the shell of hatched eggs of nest 2, while *F. falciforme* was present in the internal content of unhatched eggs of nest 1 and the shell of unhatched eggs of nest 2. Although *Fusarium* was not found inside the eggs of nest 2, its effect on egg development and hatchings could occur without direct contact with the eggs. In fact, some species of *Fusarium* are known to produce volatile mycotoxins or other metabolites that affect adjacent eggs influencing embryo development [34, 35]. Our results strongly suggest that *Fusarium* spp. did not represent the only cause of the mortality of eggs in nest 1 since the two genera of fungi were found in both nests. Therefore, a difference in bacterial abundance of the two nests might have a more predominant role in hatching success.

### Bacteriological Analysis

Interestingly, the search for bacterial pathogens showed different bacteria present inside the nests (Table 2).

**Table 2:** Bacterial genera isolated from nests 1 and 2. Bacteria were identified by sequencing the 16S rRNA gene of one isolate chosen as representative of a determined morphology on the agar media used

Nest	Sample	Pseudomonas sp.	Bacillus sp.	Lysinibacillus sp.	Brucella sp.	Vibrio sp.
	Sand outside	–	+	–	–	–
1	Sand inside	+	+	–	–	–
	Shells of unhatched eggs	+	–	–	+	–
	Inner membranes of unhatched eggs	+	–	–	+	–
2	Sand inside	–	+	+	–	–
	Shells of unhatched eggs	–	+	+	–	–
	Inner membranes of unhatched eggs	–	–	–	–	–
	Shells of hatched eggs	+	–	–	–	–

The most striking feature was the presence of *Pseudomonas* in all the samples analyzed from nest 1 (sand, eggshells, and inner membranes). Differently, *Pseudomonas* was never isolated from sand, eggshells, and inner membranes from nest 2 and from the sand collected outside of both the nests. It was isolated only from fragments of the hatched eggs of nest 2, suggesting colonization after the hatching event. Even if these analyses are not quantitatively supported, we could surmise that *Pseudomonas* could have affected egg development. Indeed, *Pseudomonas* has been frequently associated with the failure of hatchings in several turtle species [14, 36, 37]. As described below, 16S rRNA gene metabarcoding confirmed that the *Pseudomonadaceae* family was abundant in nest 1, even if it was also found in the sand outside the nests. Sand from the two nests and sand outside contained *Bacillus* sp., which is commonly found in the soil. The sand of nest 2 also contained *Lysinibacillus* sp. Some species of *Lysinibacillus* produce secondary metabolites that act as very potent antimicrobial compounds [38]; thus, its presence in nest 2 could have had a role in protecting the development and hatching of the eggs. The second striking difference was the detection of *Brucella* only in nest 1. *Brucella* is an opportunistic pathogen present in various environments and is becoming a growing cause of serious infections [39]. It has been isolated from the eggs of sea turtle nests in other reports [14, 40, 41]. Its role in the failure of hatchings can be surmised, since 16S rRNA gene metabarcoding revealed its presence in all samples, except in the sand external to the nest; thus, we hypothesize that it derives from the mother. To our surprise, we have not isolated *Salmonella*, *Aeromonas*, *Citrobacter*, and *Vibrio*, which have been frequently isolated from the egg interior of unhatched *C. caretta* sea turtle eggs [8, 9, 14].

### Microbial Composition and Biodiversity by 16S rRNA Gene Metabarcoding

**Fig. 2:**
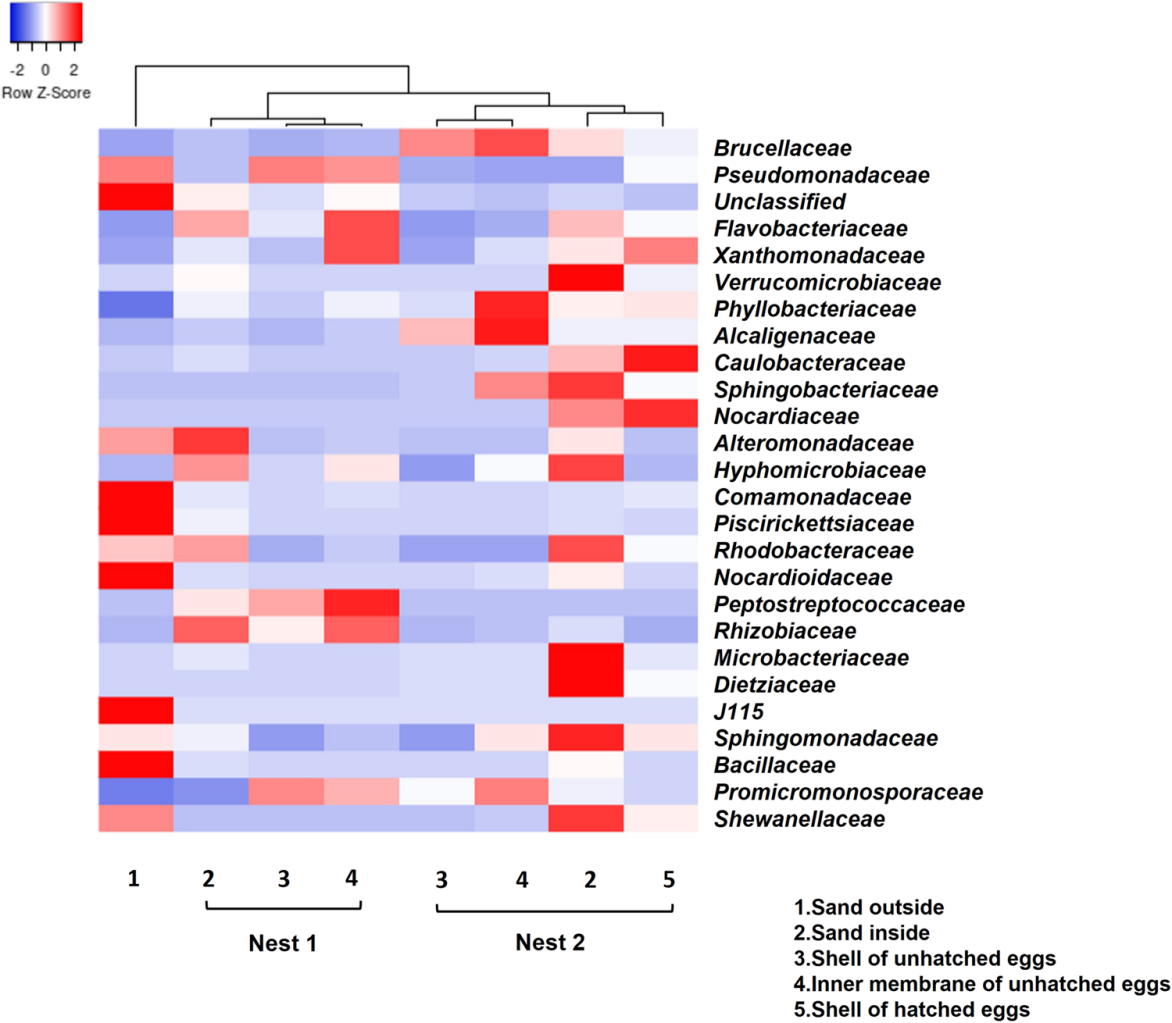
Heatmap of the 25 most abundant bacterial families generated by the “complete linkage” calculation and using Spearman’s rank correlation. A range of colors, from blue to red, indicates the relative values of the abundance of each family

High-quality reads (125,568) from the 320,073 raw reads were obtained. After filtration, denoising, and merging, 1688 ASVs (amplicon sequence variants) were identified using the suite QIIME2 (Table S1, Supplementary material). The rarefaction curves (Fig. S1, Supplementary Material) based on the comparison of ASVs abundances and the number of sequences showed that the analyses performed were representative of the communities under investigation, as confirmed by Good’s coverage index (an average of 1 for all the samples) (Table S1, Supplementary material). ASVs were classified at different taxa levels: phylum, class, order, and family (Fig. S2, Supplementary Material). The most abundant phyla in both nests were Proteobacteria, Bacteroidetes, Verrucomicrobia, Actinobacteria, and Firmicutes. Bacteria belonging to other phyla (such as Fusobacteria, Acidobacteria, Chloroflexi) were minor components and were not present in all samples. To the best of our knowledge, only one study has been carried out on the microbiota of sea turtle eggs of the species *C. caretta* which is mainly dominated by Proteobacteria, Actinobacteria, Firmicutes, Bacteroidetes, and Verrucomicrobia [24], as we found in our study with differences in the percentages of the phyla. The heat map of the bacterial families (Fig. 2) showed that the external sand was apart from all the samples of both nests, suggesting an active role of the sea turtle eggs in shaping the microbial community of the nesting site [17]. The external sand differed from the sample sands inside the two nests and showed a greater diversity, as suggested by the different ASVs found in each type of sample (Table S1, Supplementary material). Indeed, the external sand (813) contained at least 2.5-fold more ASVs than other sand samples (306 and 197 in sand inside nest 1 and nest 2, respectively). Furthermore, the Shannon-Wiener diversity index (*H*′) was 4.38 ± 2.15 for sand inside the nests and 7.93 for the external sand (Table S1, Supplementary material). This aspect could be ascribed to the environmental conditions (incubation, temperature, and humidity) of the nesting chambers that could favor the colonization of specific bacterial strains and discourage others. To our surprise, the heatmap showed that all the other samples clustered into two main groups, one corresponding to the samples of nest 1, and the other one to nest 2, again strongly suggesting an active effect of the eggs on the surrounding microbial communities of the nest.

### Bacterial Composition of Sand Samples

**Fig. 3:**
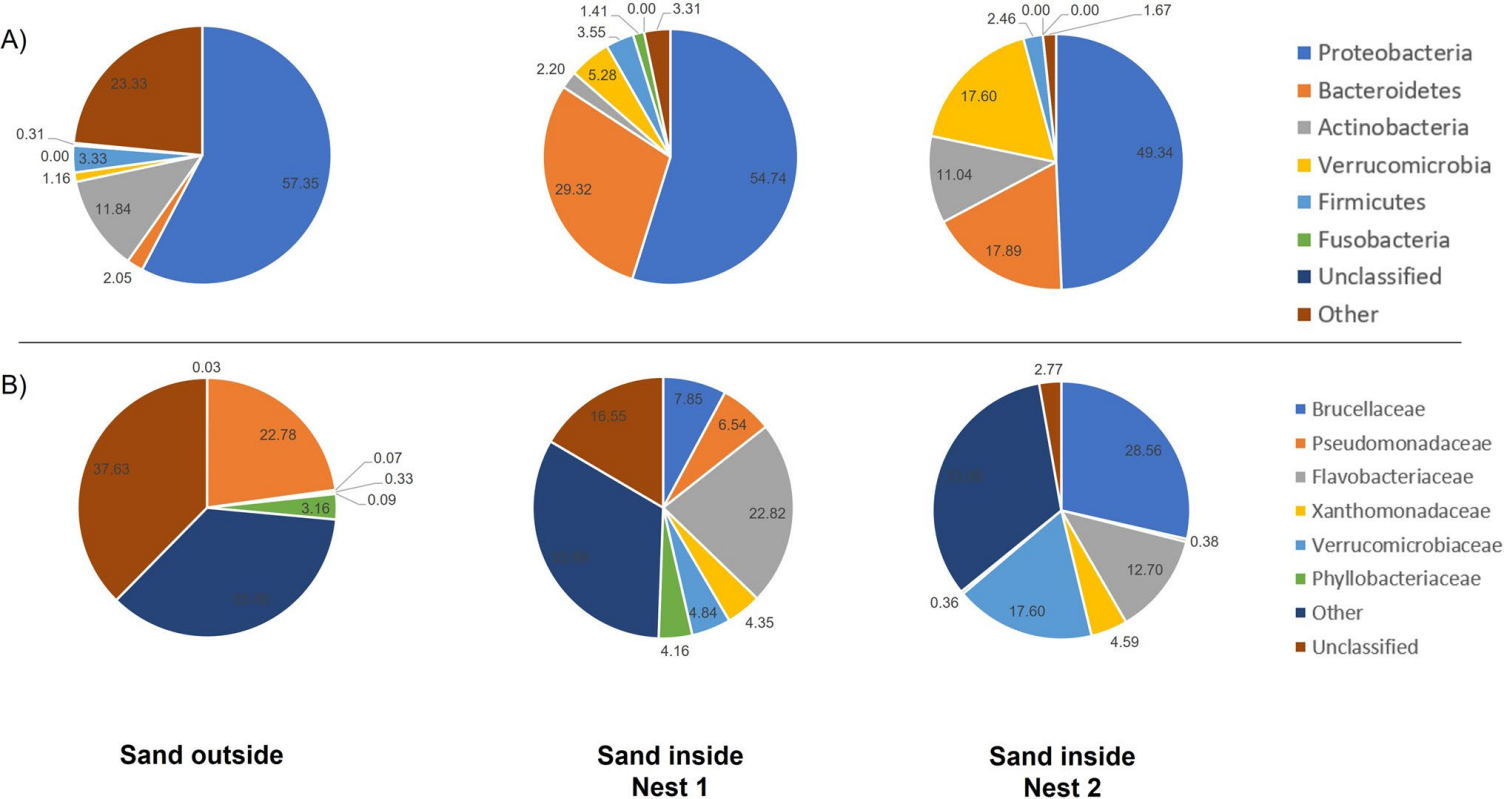
Phylum level (**A**) and family level (**B**) nest sand microbiota assortment. Pie chart showing the relative abundance of bacterial ASVs taxonomically classified at phylum and family level in sand samples.

In the sand outside the nests, a greater diversity of the low abundant bacterial taxa than in the sand collected inside the two nests was registered. The minor bacterial components are indicated as other in Fig. 3.

**Fig. 4:**
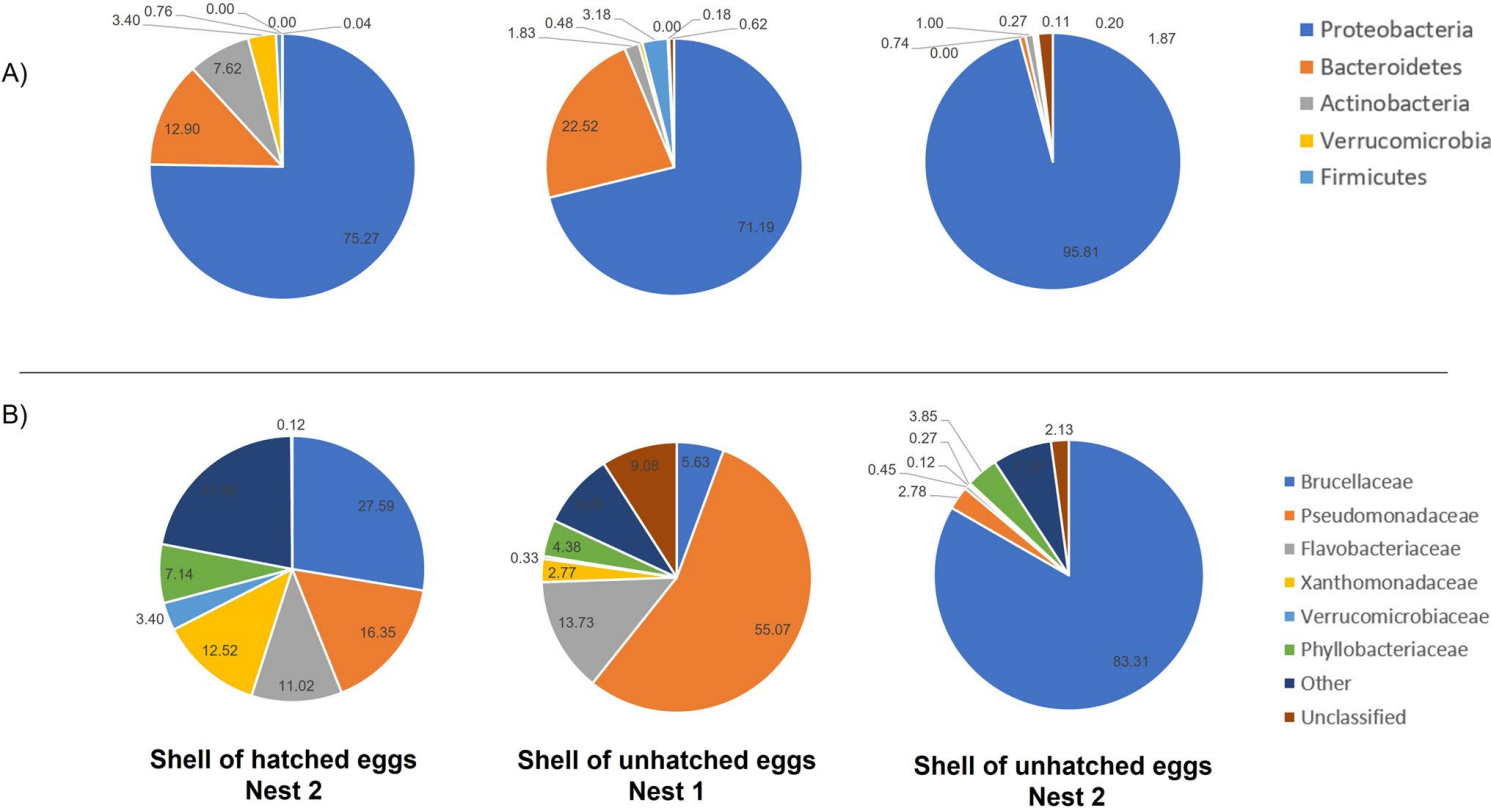
Phylum level (**A**) and family level (**B**) nest sand microbiota assortment. Pie chart showing the relative abundance of bacterial ASVs taxonomically classified at phylum and family level in the eggshell samples

Proteobacteria is the predominant phylum in the microbiota of all the sand samples (49.34–57.35%), in accordance with recent reports [24, 42]. Sand outside the nests and that one inside nest 1 contained *Pseudomonadaceae* and *Phyllobacteriaceae* families that were completely absent in the sand inside nest 2; sand inside both nests was more similar to each other, as foreseen by heatmap analysis; both nests contained *Flavobacteriaceae*, *Verrucomicrobiaceae*, *Brucellaceae*, and *Xanthomonadaceae*. This finding confirms that *Pseudomonas*, found by microbiological assay from nest 1 and sand outside, could be responsible for the failure of hatchings. We can infer that it was present in the beach sand, and it was able to colonize only samples of nest 1 because of environmental humidity and to penetrate the eggs of nest 1; in fact, it was abundant also in the shells and the inner membrane of the eggs of nest 1 (Fig. 4). As reported above, *Pseudomonas* has been frequently associated with the failure of hatchings in several turtle species [14, 36, 37]. The role of *Phyllobacteriaceae* is difficult to predict since a few reports describe its presence upon lipopolysaccharide instillation [43] and its increase in patients affected by esophageal squamous cell carcinoma [44]. Differently from the sand outside the nests, both the sands inside the nests were enriched with Bacteroidetes (29.3 and 17.89%, respectively) indicating a maternal transmission during the egg passage through the oviduct or cloaca, as already suggested [45]. Indeed, Bacteroidetes are usually less abundant in the sand and on the eggshells than inside the eggs and the cloacal samples [15, 20, 46]. Interestingly, a large abundance of Actinobacteria was registered in the sand of nest 2 (11.04%) and outside of the nests (11.84%) with respect to the sand of nest 1 containing only 2.20%. Actinobacteria produce a plethora of biologically active secondary metabolites with antibacterial and antifungal activity; thus, we could hypothesize that some elements of the nest inhibited their growth with the resulting reduction of protection of the eggs from microbial attacks [15].

Each sand sample featured specific phyla; Fusobacteria was present only in the sand inside nest 1, Verrucomicrobia was highly represented in the sand of nest 2, while the sand outside the nests contained Acidobacteria, Chloroflexi, Gemmatimonadetes, and Nitrospirae (2.87–8.76%) that were almost absent inside both nests. Usually, Fusobacteria represent a dominant phylum in the microbiota of vertebrates that feed on carrion, i.e., alligators and vultures [20, 47], and we could hypothesize that its abundance inside nest 1 was due to a premature egg death that created the proper environmental conditions for its colonization.

### Bacterial Content of Eggshells

In this study, two types of eggshells were analyzed: those deriving from the unhatched eggs, found in both the nests and fragments of eggshells found inside nest 2 after digging. These latter could derive from healthy sea turtles after the hatching event. Proteobacteria are the predominant phylum (71.19–95.81%) in turtle eggshells (Fig. 4).

As sands contained less abundance of Proteobacteria, we could surmise that bacteria belonging to this phylum have found proper growth conditions in the nests or that a maternal influence occurred. Studies of the microbiota of turtle nests have generally reported similar, but not identical, results to ours. For example, Proteobacteria, even if represented the predominant phylum, were more abundant in our study than in other reports on *C. caretta* [24] and *Eretmochelys imbricata* nests [15]. Among Proteobacteria, the *Brucellaceae* family entirely colonized the shells of the eggs of nest 2, while *Pseudomonadaceae* the nest 1. Differently, the shells of the hatched eggs showed a homogeneous bacterial distribution with a reduction of the *Brucellaceae* and *Pseudomonadaceae* families. These results strongly suggest that different bacteria affected egg development in the two nests. Although we cannot rule out that the bacterial colonization of the fragments of eggshells occurred after the hatchings in which the shells could have lost their active and protective role, together with the loss of the antimicrobial molecules inside the yolk, we suppose that *Pseudomonas* colonization derived from the sand while *Brucella* could be transmitted by the mother.

*Verrucomicrobiaceae* and *Flavobacteriaceae* were present in the eggshells of the hatched eggs of nest 2 and the unhatched eggs of nest 1. The *Verrucomicrobiaceae* family encodes a wide variety of glycoside hydrolases, sulfatases, peptidases, carbohydrate lyases, and esterases that could be used to metabolize released nutrients [46]. *Flavobacteriaceae* are commonly associated with urban environments, sewage-polluted waters, and stormwater since many members of this family need environments containing complex carbon compounds [48].

### Bacterial Content of Inner Membranes of the Unhatched Eggs

**Fig. 5:**
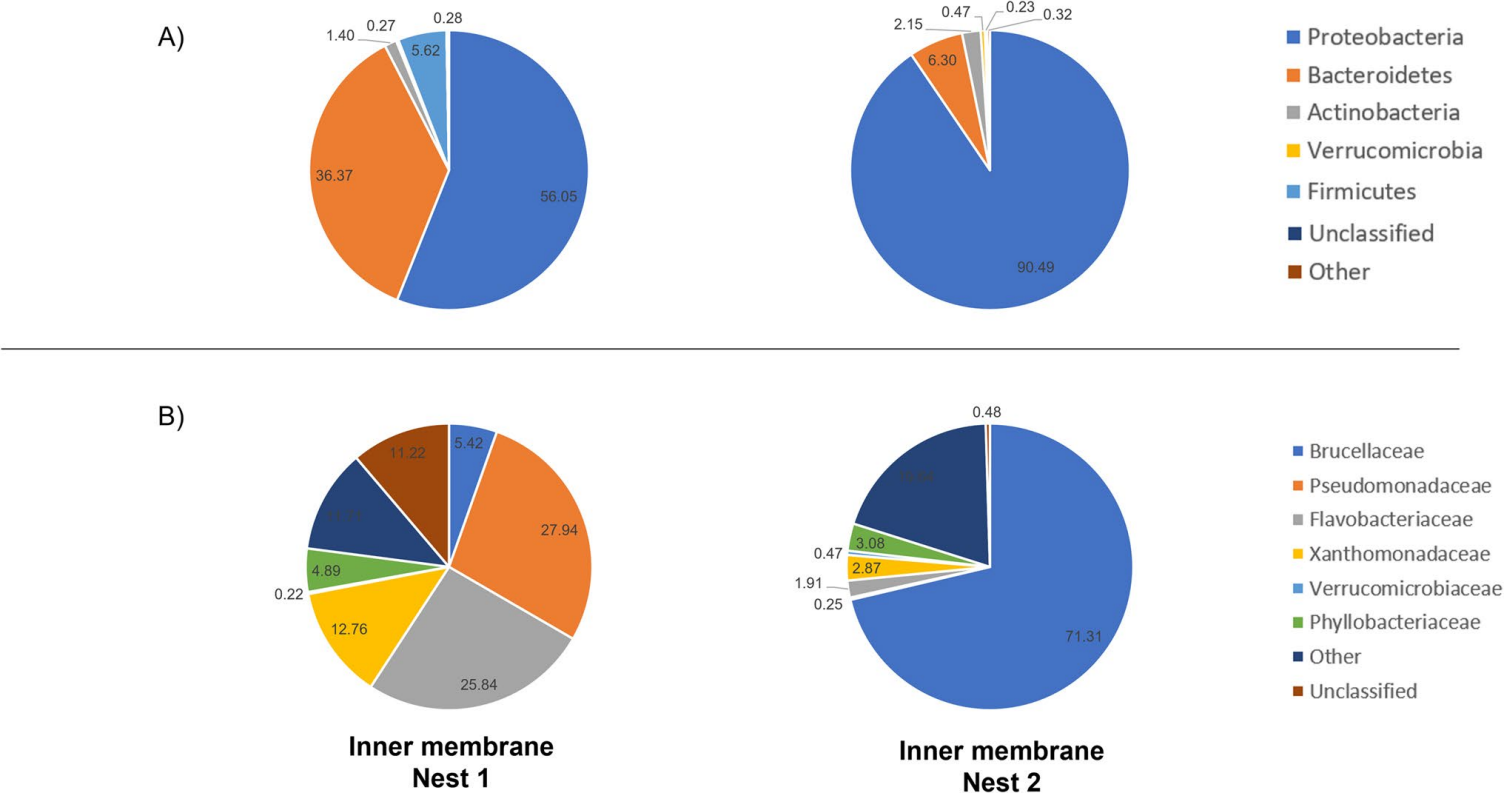
Phylum level (**A**) and family level (**B**) nest sand microbiota assortment. Pie chart showing the relative abundance of bacterial ASVs taxonomically classified at phylum and family level in the inner membranes of the eggs samples

At the phylum level, the most evident differences were the large abundance of Bacteroidetes and Firmicutes in the inner membrane of eggs of nest 1; indeed, Proteobacteria, Bacteroidetes, Firmicutes, and Actinobacteria were found in nest 1 while Proteobacteria, Bacteroidetes, Actinobacteria, and Verrucomicrobia in nest 2 (Fig. 5). Firmicutes and Bacteroidetes are found in the maternal oviduct and are less represented in the sand and seawater [21, 45]. In addition, Bacteroidetes and Firmicutes are the most represented phyla in animal and human gut microbiota [19, 20, 49]. Therefore, our hypothesis is that possible municipal wastewater could have contaminated nest 1, favoring the proliferation of Firmicutes and Bacteroidetes and disfavoring the normal proliferation of Actinobacteria and Verrucomicrobia, bacterial phyla typical of the sandy environment [50]. Thus, their presence in the inner membranes of the unhatched eggs could derive from the contaminated sand.

At the family level, analysis of the inner membranes of the unhatched eggs confirmed different colonization of the eggs, *Pseudomonadaceae* and *Flavobacteriaceae* (28% and 26 %) in nest 1 and *Brucellaceae* in nest 2 (71%). Bacteroidetes, including the *Flavobacteriaceae* family, and some families belonging to Firmicutes, such as *Peptostreptococcaceae*, *Lachnospiraceae*, and *Clostridiaceae*, are considered fecal markers, especially in humid growth conditions [51, 52]. *Flavobacteriaceae* are commonly associated with urban environments, sewage-polluted waters, and stormwater since many members of this family need environments containing complex carbon compounds [48]. *Brucellaceae* has been isolated in sea turtles with conjunctivitis infection [53] and in the nest of *Chelonia mydas* [41] and *Caretta caretta* turtles [14]. Members of the genus *Brucella* have recently been identified in several species of cetaceans and pinnipeds, and a *Brucella* species was isolated from the aborted fetal tissue of bottlenose dolphins along the California coast [54]. Thus, we surmise that 59% of the unsuccessful hatching of the eggs of nest 2 could be associated with this bacterial genus.

## Conclusions

The analyses of the microbial community in *C. caretta* nests could be used a useful tool to preserve the conservation of the species and increase hatching success. The present study is based on the analysis of two nests of *C. caretta* on the beach of Guitgia of Lampedusa, with a different success rate of hatching: 0% for nest 1 and 59% for nest 2. Combining the results of this report with our previous work conducted on nests from other sites, our findings suggest a crucial role for the sand and show that each nest has its microbial profile. Indeed, the sand inside the nests displayed a lower bacterial diversity than the sand outside, and different bacterial phyla could be responsible for the hatching failure of both nests. Although we cannot rule out the involvement of *Fusarium*, in this study, we considered *Pseudomonas* and *Brucella* the key players governing the hatching fate, with *Pseudomonas* deriving from the sand of the beach and *Brucella* from the mother. Limitations of the microbiological culture and 16S rRNA metabarcoding have to be taken into consideration pointing out that both methods should be carried out to investigate microbial community; however, it is noteworthy that bacteria detectable by DNA-based methods could not be viable and metabolically active organisms, even if DNA is detectable. Thus, periodic and preventive monitoring of microbial content of sand and nests could be helpful for nest management and protection activities. As an example, the identification of known pathogens in the sand could lead to moving the nest to another site, eliminating some threats to the conservation of the species.

## Supplementary Information

Figure S1. Rarefaction curves on sequencing data obtained from different compartments. The number of observed characteristics (representative of the ASVs) found in each sample is reported as a function of the sequencing effort. The asymptotic trend of the curves indicates that the number of readings generated is representative of the entire community. (PNG 988 kb)

High Resolution Image (TIFF 26515 kb)

Figure S2. Relative abundance (%) of phyla (A), class (B), order (C), and 25 most abundant families (D) detected in the sand, fragments of eggshells of hatched and unhatched eggs, and inner membrane of sea turtles’ eggs of two nests. (PNG 2313 kb)

High Resolution Image (TIFF 16897 kb)

Table S1. Bioinformatic analyses of obtained reads: filtering, denoising, and merging results. (PDF 153 kb)

## Data Availability

The datasets analyzed during the current study are available in the GenBank database with the accession numbers OM857961-OM857964; OM860305-OM860307; OM860310, OM860311; OM860313-OM860315; OM860317; OM860320; OM860321 and BioProject ID: PRJNA804141.
