# Supplementary figures and images for: Correlation Between Microbial Community and Hatching Failure in Loggerhead Sea Turtle Caretta caretta
Source: Microb Ecol. 2023 Feb 20;86(3):1923–33. doi: 10.1007/s00248-023-02197-8 (PMC10497424; doi:10.1007/s00248-023-02197-8)

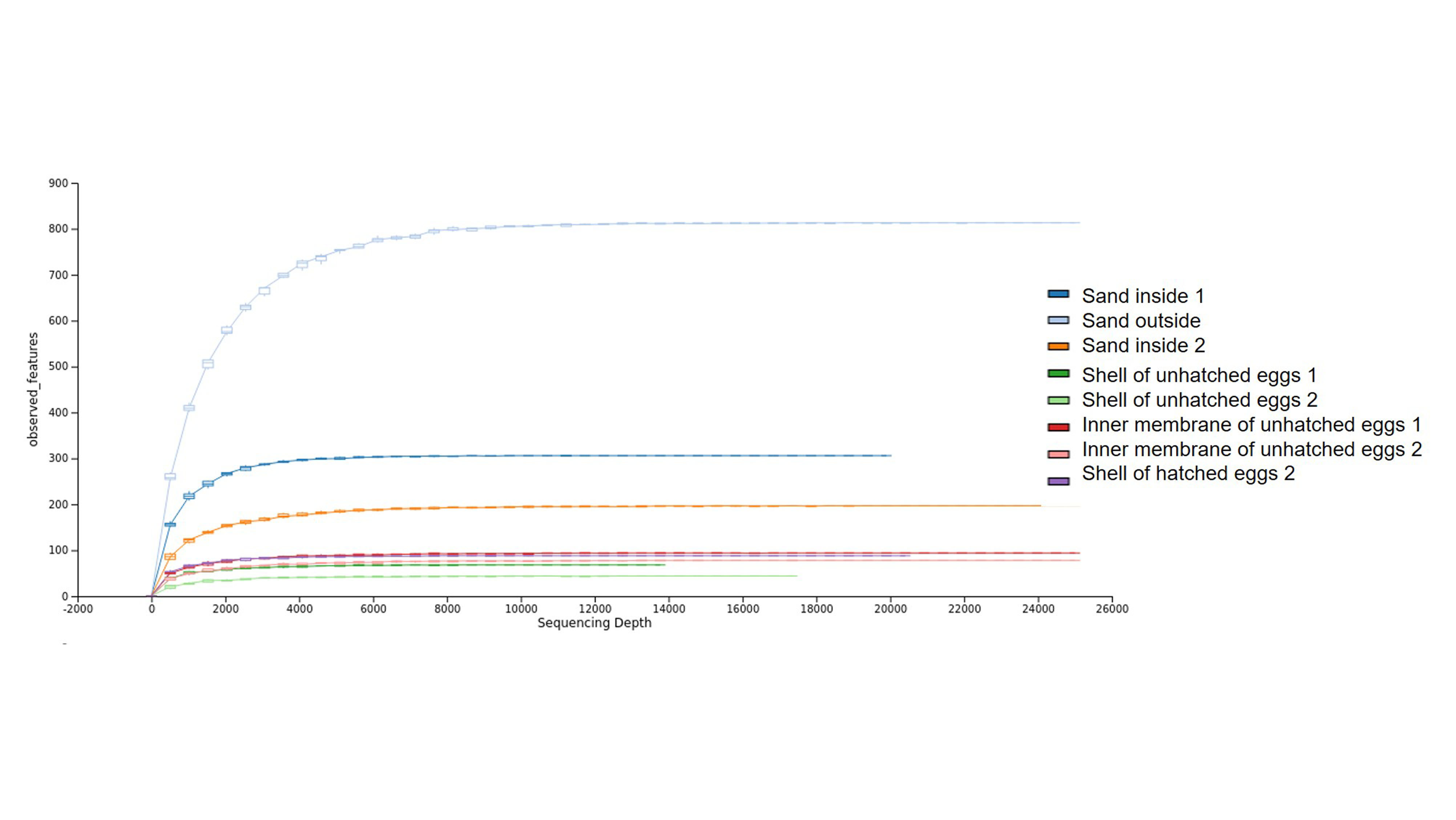

Supplement: Supplementary file 1 — Rarefaction curves on sequencing data obtained from different compartments. The number of observed characteristics (representative of the ASVs) found in each sample is reported as a function of the sequencing effort. The asymptotic trend of the curves indicates that the number of readings generated is representative of the entire community. (PNG 988 kb) [file 248_2023_2197_Fig6_ESM.png]

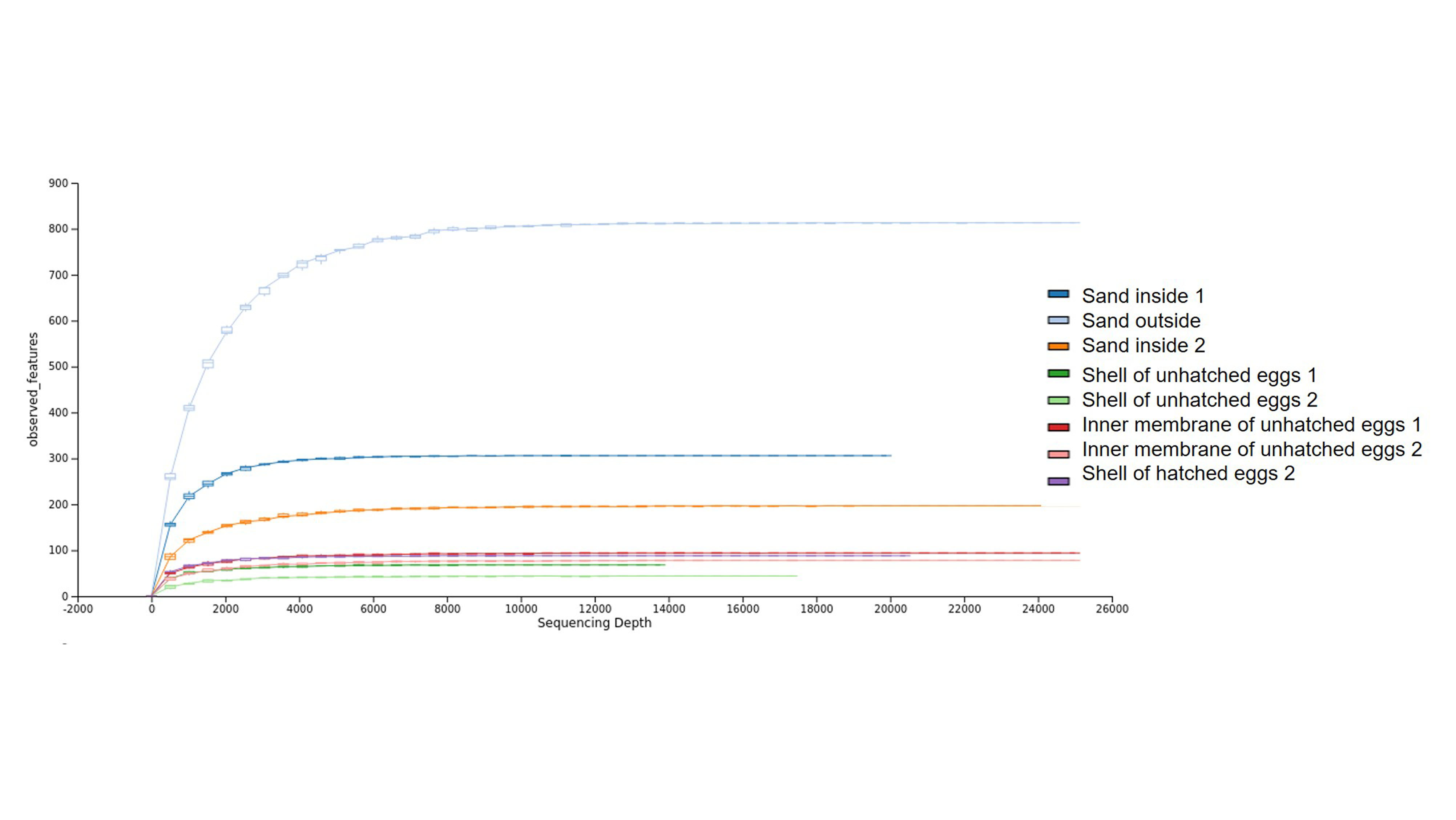

Supplement: Supplementary file 2 — High Resolution Image (TIFF 26515 kb) [file 248_2023_2197_MOESM1_ESM.tiff]

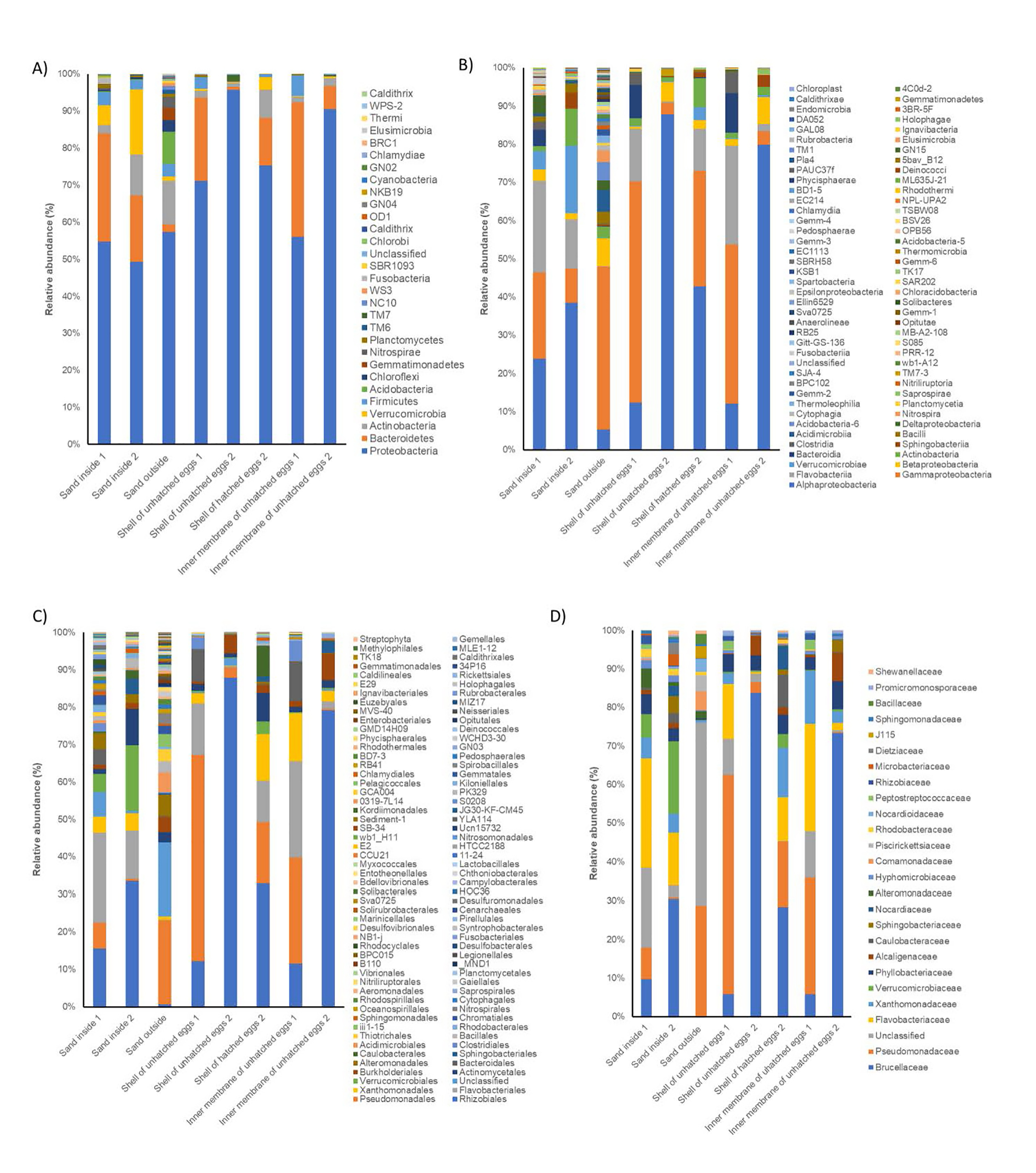

Supplement: Supplementary file 3 — Relative abundance (%) of phyla (A), class (B), order (C), and 25 most abundant families (D) detected in the sand, fragments of eggshells of hatched and unhatched eggs, and inner membrane of sea turtles’ eggs of two nests. (PNG 2313 kb) [file 248_2023_2197_Fig7_ESM.png]

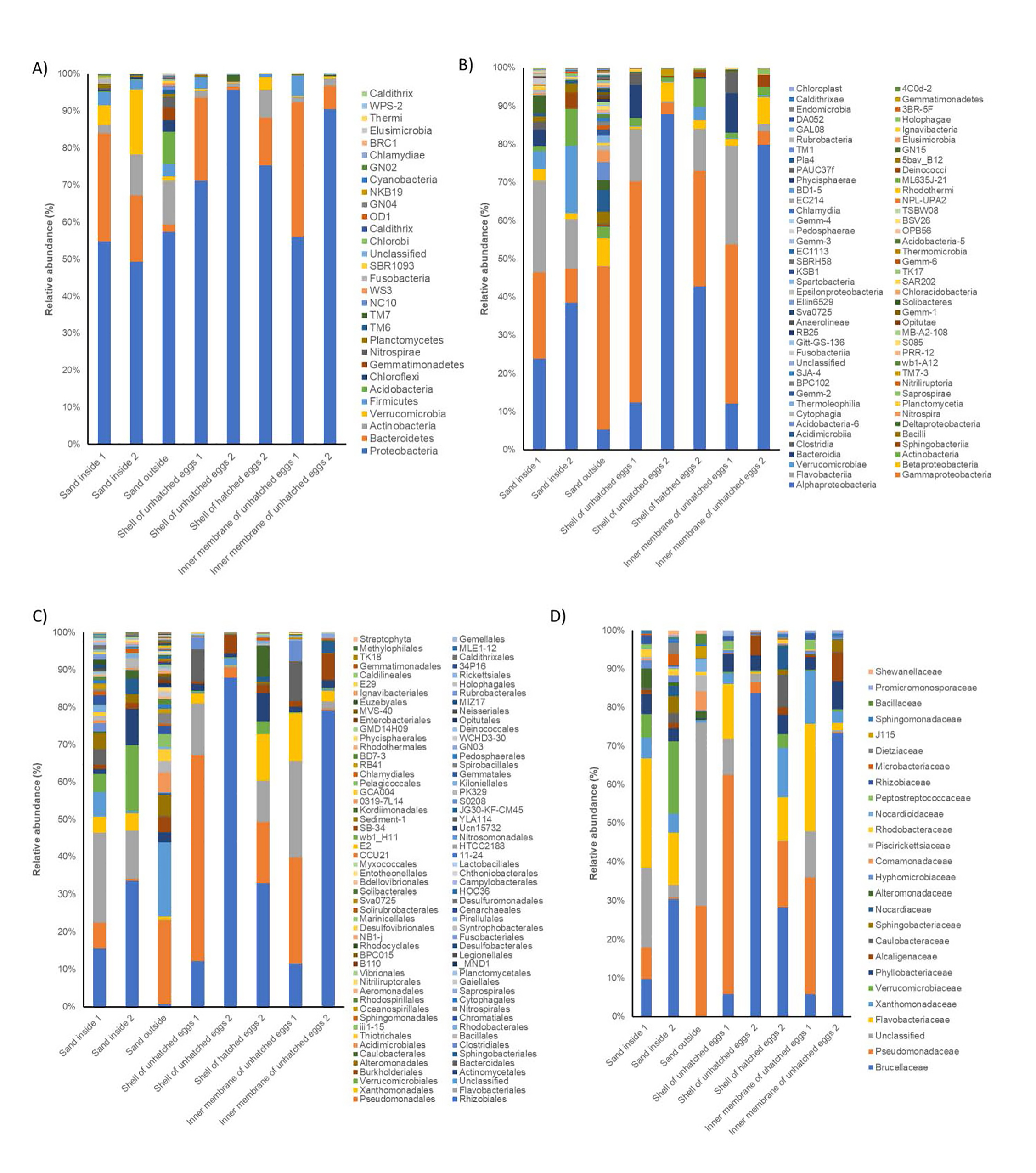

Supplement: Supplementary file 4 — High Resolution Image (TIFF 16897 kb) [file 248_2023_2197_MOESM2_ESM.tiff]
