# Supplementary material for: Correlation Between Microbial Community and Hatching Failure in Loggerhead Sea Turtle Caretta caretta
Source: Microb Ecol. 2023 Feb 20;86(3):1923–33. doi: 10.1007/s00248-023-02197-8 (PMC10497424; doi:10.1007/s00248-023-02197-8)
Supplement: Supplementary file 5 — Bioinformatic analyses of obtained reads: filtering, denoising, and merging results. (PDF 153 kb) [file 248_2023_2197_MOESM3_ESM.pdf]

Correlation between microbial community and hatching failure in loggerhead sea turtle *Caretta caretta*

Fanny Claire Capri[1], Elena Prazzi[2], Giulia Casamento[3], Delia Gambino[4], Giovanni Cassata[4], Rosa Alduina[1*]

[1] Dipartimento Scienze e Tecnologie Biologiche, Chimiche e Farmaceutiche, Viale delle Scienze, University of Palermo, 90133 Palermo, Italy

[2] Legambiente Sicilia- Ente Gestore Riserva Naturale Orientata Isola di Lampedusa, Via Vittorio Emanuele, 25, 92031 Lampedusa (AG), Italy

[3] Legambiente Sicilia- Ente Gestore Riserve Naturali, via Paolo Gili,4, 90138 Palermo (PA), Italy

[4]Istituto Zooprofilattico Sperimentale della Sicilia "A. Mirri", 90129 Palermo, Italy

*** Correspondence:**
Rosa Alduina

valeria.alduina@unipa.it

Supplementary Table

Table S1. Bioinformatic analyses of obtained reads: filtering, denoising, and merging results. Diversity indices of the samples used in this study: Chao1 and ACE are abundance-based richness estimator; H' is the Shannon-Weiner diversity index.

Samples	Input	Filtered	Percentage of input passed to filter (%)	Denoised	Merged	Percentage of input merged (%)	Non-chimeric	Percentage of input non-chimeric	Good's Coverage	ASVs	ACE	H'	Chao 1
Sand outside the nests	65651	43893	66.86	42640	39466	60.11	39280	59.83	1	813	813	7.93	813
Sand inside 1	34719	23183	66.77	22663	21861	62.97	21188	61.03	1	306	306	6.73	306
Sand inside 2	51994	34330	66.03	33390	32090	61.72	31811	61.18	1	197	197	4.81	197
Shell of unhatched eggs 1	23723	16169	68.16	15823	15148	63.85	13989	58.97	1	68	68	3.72	68
Shell of unhatched eggs 2	29233	19572	66.95	19384	19140	65.47	18036	61.7	1	44	44	1.31	44

Shell of hatched Eggs 2	32368	21638	66.85	21485	21115	65.23	20699	63.95	1	88	88	4.14	88
Inner membranes of unhatched eggs 1	38431	26342	68.54	26076	25726	66.94	25312	65.86	1	94	94	4.10	94
Inner membranes of unhatched eggs 2	43954	30771	70.01	30484	29732	67.64	27607	62.81	1	78	78	2.31	78
